# Are Electrocochleographic Changes an Early Sign of Cochlear Synaptopathy? A Prospective Study in Tinnitus Patients with Normal Hearing

**DOI:** 10.3390/diagnostics12040802

**Published:** 2022-03-25

**Authors:** Kuan-Chung Ting, Chia-Chen Chang, Chii-Yuan Huang, Yu-Fu Chen, Yen-Fu Cheng

**Affiliations:** 1 Department of Otolaryngology—Head and Neck Surgery, Taipei Veterans General Hospital, Taipei 112, Taiwan; kctingmd@gmail.com (K.-C.T.); dopod0635@gmail.com (C.-Y.H.); 2 Department of Otolaryngology, Taipei Veterans General Hospital, Taoyuan Branch, Taoyuan 330, Taiwan; 3 Faculty of Medicine, National Yang Ming Chiao Tung University, Taipei 112, Taiwan; 4 Institute of Clinical Medicine, College of Medicine, National Yang Ming Chiao Tung University, Taipei 112, Taiwan; 5 Department of Speech-Language Pathology and Audiology, National Taipei University of Nursing and Health Sciences, Taipei 112, Taiwan; jennychung2933@gmail.com (C.-C.C.); yufuchen@ntunhs.edu.tw (Y.-F.C.); 6 Department of Medical Research, Taipei Veterans General Hospital, Taipei 112, Taiwan; 7 Institute of Brain Science, College of Medicine, National Yang Ming Chiao Tung University, Taipei 112, Taiwan

**Keywords:** hidden hearing loss, ECochG, tinnitus, cochlear synaptopathy

## Abstract

The mechanism of tinnitus accompanied by a normal audiogram remains elusive. This study aimed to investigate evidence of primary neural degeneration, also known as cochlear synaptopathy, in tinnitus patients with normal hearing thresholds. We analyzed the differences in electrocochleography (ECochG) measurements between normal-hearing subjects with and without tinnitus. Forty-five subjects were enrolled in this study: 21 were in the tinnitus group, defined by chronic tinnitus of over two months’ duration with normal audiometric thresholds, and 24 were in the control group, defined by a lack of tinnitus complaints. Electrocochleograms were evoked by 1, 4, 6, and 8 kHz alternating-polarity tone bursts at sound pressure levels (SPLs) of 90–110 dB. The tinnitus group had smaller action potential (AP) amplitudes than the control group for 1, 4, 6, and 8 kHz tone bursts and showed significant amplitude reduction at 1 kHz 110 dB SPL (*p* < 0.01), 1 kHz 90 dB SPL (*p* < 0.05), and 4 kHz 110 dB SPL (*p* < 0.05). There were no significant differences in the summating potential/action potential (SP/AP) amplitude ratios across the four tested frequencies. A trend of reduced AP amplitudes was found in the tinnitus group, supporting the hypothesis that tinnitus might be associated with primary neural degeneration.

## 1. Introduction

Tinnitus is the perception of noise without an external acoustic stimulus; this phenomenon is often described as ringing or buzzing in the ears and is estimated to occur in between 4.6% and 30% of the population [1,2,3,4,5,6,7]. In approximately 1–2% of tinnitus cases, patients’ quality of life is seriously degraded, resulting in depression, social isolation, and even sleep deprivation [8]. This symptom is generally believed to be associated with some degree of hearing loss [9,10], but very little is known about the pathological mechanism underlying tinnitus with normal hearing thresholds. Damage to the synapses between afferent auditory nerve fibers and inner hair cells (IHCs), also known as “cochlear synaptopathy”, provides a possible explanation for this subset of tinnitus [11,12].

In recent years, there has been increasing interest in attempting to detect cochlear synaptopathy in humans, especially for those without obvious hearing threshold shifts. Kujawa et al. [13] first reported that in mice with noise-induced temporary hearing loss, in addition to the loss of cochlear synaptic terminals and cochlear ganglion cells on histopathological studies, the amplitude of auditory brainstem response (ABR) wave I recovered to only 40% at 32 kHz after high-sound-intensity stimulation. They found that the reduction in the number of synapses was correlated with the ABR results. On this basis, it can be reasonably inferred that objective electrophysiological techniques are a possible indicator for detecting cochlear synaptopathy in humans [8,14,15,16]. Liberman et al. [14] further utilized speech-in-noise tests and electrocochleography (ECochG) to detect potential synaptopathy in participants with different degrees of noise exposure: they divided normal-hearing college students into a high-risk group and a low-risk group based on their self-reported music exposure. The high-risk group had a poorer outcome on the speech-in-noise test and a significantly higher summating potential (SP)/compound action potential (AP) ratio than the low-risk group [14]. The SP and AP are produced in hair cells and the distal part of cranial nerve VIII. Therefore, the SP/AP ratio may have clinical value in the diagnosis of hidden hearing loss.

Very few studies have explored the SP/AP ratios on ECochG in tinnitus patients with normal hearing [17,18]. This prospective study aimed to investigate the clinical significance of the ECochG in normal-hearing subjects with and without tinnitus and to explore the correlation between ECochG results and the subjective degree of tinnitus annoyance.

## 2. Materials and Methods

### 2.1. Participants

Forty-five participants aged 20 to 65 years were included in this study. Tinnitus participants were recruited from the outpatient clinic at Taipei Veterans General Hospital from January 2018 to February 2020 if they had chronic tinnitus complaints lasting at least two months and did not have any history of otological diseases (i.e., auditory neuropathy, tumors, middle ear pathologies, mandibular joint neuralgia, etc.). The control group was recruited by advertisement or personal invitation. All participants had normal bilateral audiometric thresholds (≤25 dB HL (decibels hearing level)) at 250 Hz~8 kHz, normal impedance functions (peak pressure range ±100 daPa; static compliance between 0.3 and 1.5 mL), and distortion-product otoacoustic emissions (DPOAEs) presented at 750 Hz to 8 kHz of f2. Tinnitus participants were required to complete the Mandarin version of the Tinnitus Handicap Inventory (THI) questionnaire [19] to evaluate the association between ECochG results and THI scores. All measurements were performed monaurally, and all control participants were assigned to have the right ear tested. For members of the tinnitus group with bilateral tinnitus, the ear with louder tinnitus was chosen as the test ear. If the loudness of tinnitus was equal in both ears, the right ear was selected for testing. The study was approved by the Institutional Review Board (IRB No. 2017-11-005BC) of Taipei Veterans General Hospital. All procedures were performed in accordance with the relevant guidelines and regulations. Informed consent was obtained from all participants.

### 2.2. Procedures

Prior to all tests, otoscopy was used to inspect the patency of the external auditory canal and the intactness of the eardrum. Tympanometry was carried out with a GSI TympStar Pro (Grason-Stadler Inc., Eden Prairie, MN, USA) using a 226 Hz probe tone, and type A subjects were screened out. Pure-tone air conduction thresholds were measured using a GSI AudioStar Pro (Grason-Stadler Inc., Eden Prairie, MN, USA) audiometer with TDH-50P headphones at octave frequencies between 250 Hz and 8 kHz, and the average hearing threshold at 500 Hz, 1 kHz, and 2 kHz was defined as the pure-tone average (PTA). Bone conduction thresholds were assessed at frequencies from 500 Hz to 4 kHz using a bone vibrator, and air–bone gaps were ≤10 dB at the test frequencies. DPOAEs (Natus Bio-logic Scout Sport) were measured by two primers, f1 and f2, with intensities of L1 = 65 dB sound pressure level (SPL) and L2 = 55 dB SPL. The f2/f1 ratio was set at 1.22. When the signal-to-noise ratio was ≥6 dB, it indicated that the outer hair cell function was normal.

### 2.3. Electrophysiological Recording

For electrophysiological recording, a Bio-logic auditory evoked potential system (Navigator 7.0.0; Natus, Pleasanton, CA, USA) was used to generate the stimuli and collect the data. The electrode montage for one-channel ECochG consisted of an active electrode on the upper forehead, an electrode on the contralateral mastoid as a ground, and a gold-foil-tipped electrode in the subject’s ear canal as a reference. Impedances between electrodes were controlled at <5 kΩ in all recordings. ECochG was evoked by 1, 6, and 8 kHz tone bursts (80, 90, and 110 dB SPL) and 4 kHz tone bursts (80, 90, 100, and 110 dB SPL) whose polarity alternated at a rate of 11.1 cycles/sec. The analysis focused on the 4 kHz results because the largest reduction in ABR wave I amplitude was found at 4 kHz [16]. The duration of each tone burst was 2 msec, with a 0.5 msec rise and fall time for all frequencies. A bandpass filter with a window of 10 to 1500 Hz was applied, and each waveform was averaged over 1500 sweeps and recorded at least twice to confirm the reproducibility. Data were excluded if the subjects had no obvious response waveforms at 80 or 90 dB SPL. All subjects were placed in the supine position and required to relax or sleep in a quiet room for approximately 1.5 h during the test. If the subjects indicated that they could not continue cooperating during the measurement, the waveform recording was also stopped. Two examiners determined the peaks and baseline of AP and SP. The amplitudes of AP and SP were defined as the vertical distance between the peak and the baseline [14]. The onset of SP, which was also regarded as a small shoulder preceding the AP, was selected as the baseline. The AP was identified as the largest peak, which occurred 1.5~2 ms after stimulus onset.

### 2.4. Statistical Analysis

All data were first entered into an Excel spreadsheet and imported into the statistical software IBM SPSS Statistics 20 for further analysis. Descriptive statistics were presented in the form of minimum, maximum, average, and standard deviation to investigate the individual subjects’ data, including sex, age, PTA threshold, SP amplitudes, AP amplitudes, and SP/AP ratios on ECochG at each frequency. The intensity and THI scores of the tinnitus group were also considered. An independent-sample t test was used to test the relationship of the observed variables between two groups. Pearson product-moment correlation was used to compare the differences between THI scores and the results of all electrophysiological tests in the tinnitus group. All comparisons were two-tailed, and a *p* value of less than 0.05 was considered statistically significant.

## 3. Results

### 3.1. Characteristics of Study Subjects

**Figure 1:**
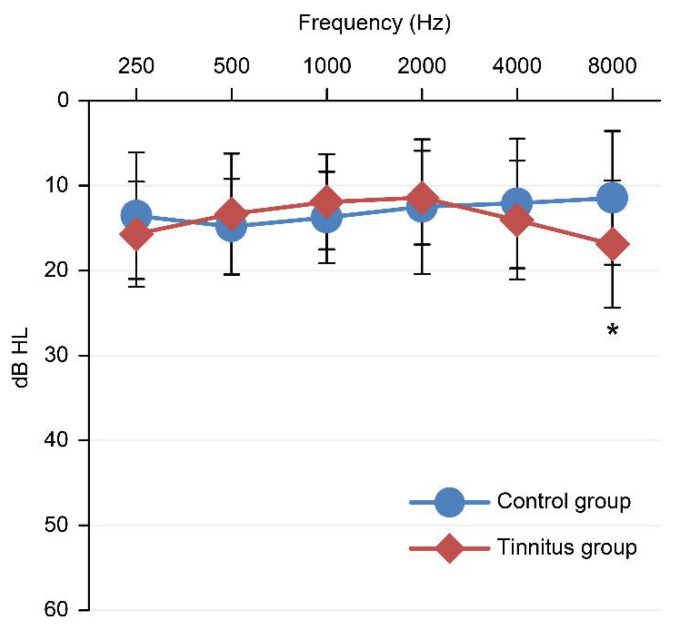
Hearing threshold curves of the two groups of test ears. Error bars show 95% confidence intervals; * p < 0.05.

A total of 45 subjects participated in this study (Table 1). The tinnitus group consisted of 21 individuals with tinnitus (18 women, 3 men, mean age 43.2 ± 10.9 years, totaling 21 ears), and the control group consisted of 24 individuals without tinnitus (20 women, 4 men, mean age 36.9 ± 7.5 years, totaling 24 ears). There was no significant difference in sex (*p* = 0.831) or pure-tone average thresholds (500 Hz, 1 kHz, and 2 kHz) between the tinnitus and control groups (*p* = 0.341), although the tinnitus group was slightly older than the control group. Figure 1 shows the distribution of audiometric thresholds at the standard test frequencies (250 Hz~8 kHz) for the two groups. All subjects had normal hearing thresholds (<25 dB HL), but the tinnitus group showed significant threshold elevation at 8 kHz (*p* = 0.02).

The THI was used as a subjective assessment of tinnitus perception. All subjects in the tinnitus group were subjected to the THI (*n* = 21), with a score ranging from 6 to 86, an average score of 42.8, and a standard deviation of 22.4. Most of the tinnitus subjects in this study had mild and moderate levels of tinnitus annoyance (66.6%). In addition, among the three subscales, the functional factor showed the average highest scores, followed by the emotional and catastrophic factors (Table 2 and Table 3).

### 3.2. ECochG Measurements

The means and standard deviations of SP amplitudes, AP amplitudes, and SP/AP ratios of the tested ears in each group at each frequency and stimulation are presented in Table 4. Each waveform was recorded at least twice to confirm reproducibility. If there were no obvious response waveforms for a certain variable, the subsequent lower stimulus levels were not recorded. Dashes indicate that no data were available. In this study, the reproducibility rate of AP and SP showed that as the stimulus became louder, the responses were more likely to be evoked. The AP amplitudes of 24 subjects in the normal group could be measured at each test frequency for 110 dB SPL, and 21 subjects in the tinnitus group also had identifiable AP amplitudes induced at 110 dB SPL, except at 8 kHz; 1 kHz had one missing data point. The SP peaks were recorded at high intensity (i.e., 110 dB SPL) at each test frequency in the two groups. As the stimulation intensity decreased, the SP waveforms became more difficult to identify for both groups. As a result, fewer data on the SP/AP ratio were documented.

The AP amplitudes of the tinnitus subjects were significantly reduced compared with those of the control group. Table 5 shows the differences in AP amplitude averages between the two groups. Three variables, including 4 kHz 110 dB SPL (0.35 ± 0.12 vs. 0.44 ± 0.12, *p* < 0.05), 1 kHz 110 dB SPL (*p* < 0.01), and 90 dB SPL (*p* < 0.05), showed significant differences in AP amplitudes. Although there was no significant difference in other variables, an overall trend of reduced AP amplitudes was observed in the tinnitus group compared to the control. There was no statistically significant difference in the SP/AP ratios between the two groups in any of the parameters analyzed (Table 6).

The correlations between THI scores, AP amplitudes, and SP/AP ratios in the tinnitus group at each frequency and stimuli are shown in Table 7. The results showed no significant correlations between THI scores, AP amplitudes, and SP/AP ratios at any frequency or intensity.

## 4. Discussion

Our results show a trend of AP amplitude reductions of ECochG for the tinnitus group with normal audiometric thresholds, mainly at 1 and 4 kHz, indicating a possible pathological role of cochlear synaptopathy in tinnitus. The concept of cochlear synaptopathy is described as the loss of hair cell synapses without hearing threshold elevation [13,16]. Schaette and McAlpine [8] first proposed the concept of “hidden hearing loss” to describe damage at cochlear synapses but still have normal hair cell function, and tinnitus may be a manifestation accompanied by hidden deficits. The auditory nerve fibers connected to the hair cells have different firing thresholds [8,13,20]. When the cochlea is injured, some nerve fibers with higher thresholds will cause transmission barriers to the IHCs. Although individuals’ hearing can be maintained at a normal level under these conditions, nerve fibers may be prevented from fully participating in the discharge response, resulting in a decrease in AP amplitudes. Our hypothesis was that the function of hair cells and nerve fibers would be affected by synaptopathy, which would further reduce the peripheral input, leading to the destruction of the balance of excitation and inhibition in the auditory center, thus inducing the perception of tinnitus. In line with this hypothesis, our study found that the tinnitus group’s AP amplitudes were lower than those of the control group. Therefore, there is a reasonable possibility of lesions to the synaptic connection between hair cells and nerve fibers in tinnitus subjects with a “normal hearing threshold”.

Long-term accumulation of noise damage may be the reason for significantly reduced AP amplitudes at 1 kHz and 4 kHz. Kujawa et al. [13] demonstrated in mice that exposure to 2 h of noise at 100 dB SPL was sufficient to cause a temporary threshold shift (TTS) without permanently damaging hair cells, but it also led to 50–60% irreversible loss of IHC synaptic ribbons. Although the results showed that the DPOAE and ABR thresholds of the mice returned to normal a few weeks after noise exposure, the spiral ganglion cells degenerated slowly and could last several months to years [13]. Cho et al. [21] observed that mice with a blast exposure history did not lose hair cells in the apex (low-frequency band) and middle regions of the cochlea, but the ribbon synapses connected to them were reduced. Fernandez et al. [22] also reported that exposing mice to a single, high-intensity burst accelerated the rate of synapse loss over time, leading to the spread of synaptopathy to the apex of the cochlea. Hickox and Liberman [23] found reduced amplitudes of ABR wave I and hyperacusis-like responses after noise exposure in a mouse model [23]. Interestingly, a reduction in the wave I amplitude of ABR in tinnitus subjects with normal hearing compared to matched controls was observed [8,24]. A previous study suggested that the degeneration of cochlear synapses may potentially trigger a decrease in afferent input, causing a poor level of sound tolerance in tinnitus patients [25]. Tinnitus and hyperacusis may originate from an unusual increase in the gain of the central auditory system in response to loss of peripheral signal input, as seen with cochlear synaptic damage [23,26,27,28].

There was no significant difference in the SP/AP ratio between the two groups at all frequencies and intensities in our study. The role of SP/AP in synaptopathy was explored in noise-exposed patients with normal hearing thresholds. Liberman et al. [14] found significant differences in the SP/AP ratios between subjects with high-risk and low-risk noise exposure. Ridley et al. [29] recruited 20 subjects with an average sensorineural hearing loss of 28 dB HL at 4 kHz as an experimental group. Although they observed an enhancement of the SP/AP ratio of the experimental group that increased with thresholds in noise, there was no significant difference in the SP/AP ratio between the experimental group and the normal-hearing group [29]. The latter study divided 30 subjects with normal hearing thresholds into a low-noise-exposure group and a high-noise-exposure group based on their history of noise exposure. The results indicated that lifetime noise exposure was unrelated to SP/AP. Moreover, the SP/AP ratio showed poor test–retest reliability due to the high variability of the SP [30]. This is not surprising, as SP is often difficult to induce and recognize clinically due to its complex response components compared to AP [29,31]. Some previous studies suggested that increasing the stimulus intensity might evoke a significant SP waveform [29,32]. Liberman et al. [14] used a click of 94.5 dB normalized hearing level (nHL; equivalent to 130 dB SPL) as the stimulus to evoke SP; this stimulus intensity is much higher than that in the current study. However, it must be considered that sound stimulation at approximately 80 dB nHL may be unbearable for some people [24]; therefore, the inconsistency may be related to SP’s low-replicability waveforms in our study, resulting in some missing values of the SP/AP ratio and reduced sample numbers, which could further mask any statistically significant results.

There was no correlation between objective ECochG responses and subjective THI. Our results showed that the THI scores were not correlated with AP amplitudes and SP/AP ratios. Lack of association between electrophysiological responses and THI scores also concurs with Kehrle et al. [33], who reported a statistically significant positive correlation between the degree of tinnitus annoyance and the levels of depression and anxiety, but no correlation was found between the THI scores in tinnitus patients and ABR responses with an expected prolonged latency. Granjeiro et al. [34] also reported that outer hair cell function was not correlated with tinnitus annoyance as evaluated by the THI. These results indicate that the degree of tinnitus annoyance may be related only to the patient’s cognition and psychological state and not to electrophysiological results.

It has been shown that hearing sensitivity degenerates gradually with age, starting in the high-frequency region, but there is no obvious difficulty in hearing until 60 years of age [35]. The average age of the tinnitus group in this study was 43.2 years; thus, they were slightly older than the control group. The hearing threshold of all subjects in the tinnitus group was within the normal range of ≤25 dB HL at 8 kHz, but it was still significantly different from those in the control group. It has been shown in mouse and human temporal bone studies that structural damage and a decrease in the number of cochlear synapses, even preceding changes in IHCs and auditory thresholds, occur not only after noise exposure but also with aging [15,36]. Age-induced synaptopathy in mice is also associated with reduced ABR wave I amplitude [36]. Therefore, it should be taken into account that aging may be the source of tinnitus perception.

On the other hand, the ECochG waveforms of the two groups were not much different at 8 kHz. A possible explanation is that the synapses in the high-frequency regions of tinnitus subjects have indeed been damaged. However, the amount of synaptic loss caused by aging is not adequate to reflect the changes in the ECochG waveforms. It is also worth noting that the amount of noise exposure gradually accumulates with age. In other words, age and noise exposure have a certain degree of interaction in their effects on the hidden deficits of the human auditory system. However, in this study, the tinnitus group showed statistically significant decreases in AP amplitudes at 4 kHz and 1 kHz, which are susceptible to noise exposure.

The study has some limitations. Our study suffers from limited sample sizes because of the challenging task of recruiting subjects with tinnitus but normal hearing. The lack of a significant correlation in the SP/AP ratio between the two groups in our study may be due to the small sample size. Poor SP amplitude reproducibility limits the use of SP/AP ratios in the evaluation of tinnitus. Although immunocytochemical quantification of synapses has provided direct evidence of synaptic changes in animal studies, synaptopathy in humans cannot be confirmed by invasive procedures. Although electrophysiological experiments in mice have shown that the amplitude of the response is correlated with cochlear synaptic lesions [13,36], it is necessary to consider individual differences among subjects in human electrophysiological studies; such differences may arise from variables such as head shape, physiological noise, or interference in the test environment.

## 5. Conclusions

The AP amplitudes on ECochG in tinnitus subjects with normal hearing levels tended to be lower than those of the control group, especially at high stimulus intensities at 1 kHz and 4 kHz. Electrophysiological testing can provide supplementary evidence to evaluate the synchronous firing of neural pathways. Although ECochG can evoke a larger AP response, the difficulty in identifying the SP amplitude causes the value of the SP/AP ratio to be limited. Further studies are needed to establish a standard norm for electrophysiological responses.

## Figures and Tables

**Table 1:** Characteristics of participants in the tinnitus and control groups.

	Tinnitus (*n* = 21)	Control (*n* = 25)	*p*
Sex			
Male	3 (14.3%)	4 (16.7%)	0.831
Female	18 (85.7%)	20 (83.3%)	
Age (mean ± SD)	43.2 ± 10.9	36.9 ± 7.5	0.031 *
PTA (500 Hz, 1 kHz, 2 kHz)	12.7 ± 5.09	13.2 ± 5.03	0.341

PTA, pure-tone average; SD, standard deviation; * p < 0.05.

**Table 2:** Descriptive statistics of THI scores in the tinnitus group.

Total Scores			Functional			Emotional			Catastrophic		
M	SD	Range	M	SD	Range	M	SD	Range	M	SD	Range
42.8	22.4	6–86	16.4	9.3	0–32	13.7	10.6	0–36	12.7	4.5	2–20

M, mean; SD, standard deviation. THI is only for the tinnitus group.

**Table 3:** Disability levels of THI scores in the tinnitus group.

Total Scores	Disability Levels	*n*	%
0–16	Very mild	2	9.5
18–36	Mild	7	33.3
38–56	Moderate	7	33.3
58–100	Severe	5	23.8

**Table 4:** Descriptive statistics of the ECochG test results in the two groups.

Frequency (Hz)	Intensity (dB SPL)	Tinnitus						Control					
		*n*	SP (μV; Mean ± SD)	*n*	AP (μV; Mean ± SD)	*n*	SP/AP (Mean ± SD)	*n*	SP (μV; Mean ± SD)	*n*	AP (μV; Mean ± SD)	*n*	SP/AP (Mean ± SD)
4000	110	18	0.12 ± 0.05	21	0.35 ± 0.12	18	0.31 ± 0.12	23	0.15 ± 0.05	24	0.44 ± 0.12	23	0.34 ± 0.11
	100	16	0.11 ± 0.05	19	0.32 ± 0.13	16	0.31 ± 0.11	18	0.13 ± 0.06	23	0.34 ± 0.14	18	0.37 ± 0.14
	90	8	0.09 ± 0.05	16	0.23 ± 0.14	8	0.28 ± 0.09	10	0.11 ± 0.05	22	0.27 ± 0.13	10	0.33 ± 0.1
	80	4	0.06 ± 0.03	8	0.18 ± 0.07	4	0.38 ± 0.19	3	0.08 ± 0.03	16	0.18 ± 0.11	3	0.33 ± 0.11
1000	110	9	0.09 ± 0.03	20	0.25 ± 0.09	9	0.29 ± 0.08	15	0.11 ± 0.04	24	0.36 ± 0.16	15	0.29 ± 0.1
	90	3	0.08 ± 0.04	15	0.17 ± 0.05	3	0.34 ± 0.17	4	0.08 ± 0.03	17	0.26 ± 0.12	4	0.33 ± 0.09
	80	–	–	–	–	–	–	–	–	8	0.19 ± 0.06	–	–
6000	110	10	0.11 ± 0.07	21	0.25 ± 0.12	10	0.31 ± 0.11	14	0.13 ± 0.08	24	0.32 ± 0.16	14	0.31 ± 0.13
	90	4	0.15 ± 0.11	12	0.21 ± 0.16	4	0.43 ± 0.14	4	0.1 ± 0.03	16	0.23 ± 0.09	4	0.35 ± 0.09
	80	4	0.15 ± 0.14	5	0.22 ± 0.17	4	0.52 ± 0.12	1	0.06	8	0.16 ± 0.04	1	0.33
8000	110	8	0.09 ± 0.04	20	0.22 ± 0.07	8	0.35 ± 0.08	9	0.08 ± 0.04	24	0.23 ± 0.07	9	0.32 ± 0.1
	90	3	0.06 ± 0.02	11	0.19 ± 0.08	3	0.26 ± 0.09	–	–	14	0.16 ± 0.06	–	–
	80	–	–	1	0.09	–	–	–	–	5	0.15 ± 0.04	–	–

AP, action potential; SD, standard deviation; SP, summating potential. A dash (–) indicates that no data are available.

**Table 5:** Comparison of AP amplitudes between the two groups.

Frequency (kHz)	Intensity (dB SPL)	Tinnitus		Control		*t*	*p*
		*n*	Mean ± SD	*n*	Mean ± SD		
4	110	21	0.35 ± 0.12	24	0.44 ± 0.12	−2.373 *	0.02
	100	19	0.32 ± 0.13	23	0.34 ± 0.14	−0.46	0.65
	90	16	0.23 ± 0.14	22	0.27 ± 0.13	−0.80	0.43
	80	8	0.18 ± 0.07	16	0.18 ± 0.11	−0.03	0.98
1	110	20	0.25 ± 0.09	24	0.36 ± 0.16	−2.775 **	0.01
	90	15	0.17 ± 0.05	17	0.26 ± 0.12	−2.831 *	0.01
	80	–	–	8	0.19 ± 0.06	–	–
6	110	21	0.25 ± 0.12	24	0.32 ± 0.16	−1.69	0.10
	90	12	0.21 ± 0.16	16	0.23 ± 0.09	−0.38	0.71
	80	5	0.22 ± 0.17	8	0.16 ± 0.04	1.11	0.29
8	110	20	0.22 ± 0.07	24	0.23 ± 0.07	−0.40	0.70
	90	11	0.19 ± 0.08	14	0.16 ± 0.06	1.02	0.32
	80	1	0.09	5	0.15 ± 0.04	−1.43	0.23

SD, standard deviation. A dash (–) indicates that no data are available. * p < 0.05; ** p < 0.01.

**Table 6:** Comparison of SP/AP ratios between the two groups.

Frequency (kHz)	Intensity (dB SPL)	Tinnitus		Control		*t*	*p*
		*n*	Mean ± SD	*n*	Mean ± SD		
4	110	18	0.31 ± 0.12	23	0.34 ± 0.11	−0.85	0.40
	100	16	0.31 ± 0.11	18	0.37 ± 0.14	−1.18	0.25
	90	8	0.28 ± 0.09	10	0.33 ± 0.1	−1.16	0.27
	80	4	0.38 ± 0.19	3	0.33 ± 0.11	0.39	0.71
1	110	9	0.29 ± 0.08	15	0.29 ± 0.1	0.04	0.97
	90	3	0.34 ± 0.17	4	0.33 ± 0.09	0.18	0.87
	80	–	–	–	–	–	–
6	110	10	0.31 ± 0.11	14	0.31 ± 0.13	−0.08	0.94
	90	4	0.43 ± 0.14	4	0.35 ± 0.09	1.03	0.34
	80	4	0.52 ± 0.12	1	0.33	1.44	0.24
8	110	8	0.35 ± 0.08	9	0.32 ± 0.1	0.69	0.50
	90	3	0.26 ± 0.09	–	–	–	–
	80	–	–	–	–	–	–

SD, standard deviation. A dash (–) indicates that no data are available.

**Table 7:** Correlation analysis of THI scores with AP amplitudes and SP/AP ratios in the tinnitus group.

Frequency (kHz)	Intensity (dB SPL)	AP			SP/AP Ratio		
		*n*	r	*p*	*n*	r	*p*
4	110	21	0.05	0.819	18	−0.1	0.683
	100	19	0.08	0.76	16	0.1	0.711
	90	16	0.26	0.329	8	0.23	0.582
	80	8	−0.23	0.585	4	−0.23	0.773
1	110	20	−0.14	0.551	9	0.43	0.244
	90	15	0.03	0.91	3	0.16	0.899
	80	0	–	–	0	–	–
6	110	21	0.07	0.766	10	0.11	0.755
	90	12	−0.18	0.587	4	−0.38	0.625
	80	5	0.85	0.068	4	0.86	0.138
8	110	20	0.15	0.535	8	0.15	0.728
	90	11	−0.2	0.566	3	0.93	0.248
	80	1	–	–	0	–	–

AP, action potential; SP, summating potential; r, Pearson correlation coefficient. A dash (–) indicates that no data are available.

## Data Availability

The data presented in this study are not publicly available due to ethical constraints but are available on request from the corresponding author.
